# Belongingness among first-generation students at Stanford School of Medicine

**DOI:** 10.12688/mep.19912.1

**Published:** 2023-11-23

**Authors:** Adrian C. Delgado, Sean Dowling, Mijiza Sanchez-Guzman, Stefanie S. Sebok-Syer, Michael A. Gisondi

**Affiliations:** 1 Pediatrics, Stanford Health Care, Palo Alto, California, USA; 2 Emergency Medicine, Stanford Health Care, Palo Alto, California, USA; 3 School of Medicine, Stanford University, Palo Alto, California, USA

**Keywords:** Belonging; belongingness; medical student; medical school; first-generation; underrepresented; minority; diversity; equity; inclusion

## Abstract

**Background:**

Nationally, underrepresented minorities represent a significant proportion of the first-generation student population. These students also tend to report lower levels of belongingness compared to their peers, which may impact their wellness and be an important factor in their academic success. This study aimed to explore whether status as a first-generation student was associated with belongingness amongst medical students.

**Methods:**

In 2019, a previously validated 16-item survey was used to examine potential disparities in belongingness amongst groups of first-generation medical students. Differences between groups were assessed using a Mann-Whitney U-test for each individual item and three composite groupings of items regarding social belonging, academic belonging, and institutional support.

**Results:**

First-generation to college and first-generation to graduate school students reported lower belongingness across most individual items as well as in all three composite groups.

**Conclusions:**

Given that peer relationships and institutional support play an important role in medical student belonging, these findings represent an opportunity to address the specific needs of individuals from underrepresented groups in medicine. Doing so can support the academic and professional success of first-generation students and help close the diversity gap in medicine.

## Introduction

The case for increasing diversity and inclusion in medicine dates back several decades ^1^. In 2010, the Association of American Medical Colleges (AAMC) Roadmap to Diversity described compelling reasons for achieving medical school class diversity, emphasizing that medical students from diverse backgrounds often serve diverse patient communities in need of greater health care access later in their clinical practice ^2, 3^. As the U.S. population becomes more diverse, there is an ever-greater need for a physician workforce that reflects this diversity ^4^. However, the pipeline of minority trainees remains insufficient. U.S. medical school admissions data indicate continued disparity in the acceptance rate of non-White students, with marginal improvement of this metric over time ^5^. Individuals from groups underrepresented in medicine make up a large percentage of first-generation college students, but these students leave science and medicine at disproportionately higher rates than their peers ^6, 7^. Studies show that first-generation students lack a sophisticated knowledge of the medical school “admissions game,” underestimate their chances of admission to medical school, and underestimate their chances of completing the degree ^8– 10^. Systematic variations in belongingness experienced by first-generation students in college and medical school may impact their wellness and be an important factor in their academic success ^11^.

Goodenow and Grady defined school belonging as "the extent to which students feel personally accepted, respected, included, and supported by others in the school social environment" ^12^. While there are several theoretical models of belongingness, two are especially relevant for the purposes of this study. First, the Identity-Proximity model—an extension of Kohut’s theory—posits that one’s identity is understood through proximity to “other” groups ^13^. Through affiliation or relationships with the “other,” or lack thereof, one determines a sense of belonging. For instance, race and racism in medicine lower Black male students’ sense of belonging in medical school ^14^. Second, the ecological framework of Bronfenbrenner’s Environmental Satisfaction model puts forward that the way in which an individual interacts with others and how they experience their environment leaves them with a sense of either satisfaction and belonging or dissatisfaction and not belonging ^15^. Therefore, school belongingness can be understood as a product of the daily lived experiences of medical students as determined by their interactions with one another, their engagement with environmental factors such as diversity and inclusion programming, and their satisfaction with institutional culture and policies.

The literature provides insight into the tensions faced by groups underrepresented in medicine, including first-generation medical students, and indicates a need for closer examinations of their experiences ^16^. A better understanding of student belongingness in medical school may help improve student learning through better-designed institutional efforts to support them. Unfortunately, data about student belongingness among first-generation U.S. medical students are lacking, representing an important gap in the literature. Precise measures of belongingness are needed to understand the current environment and to evaluate for targeted interventions to improve student experiences ^17^.

This study aimed to explore whether status as a first-generation student was associated with belongingness at Stanford School of Medicine. Exploring the prevalence of this outcome creates potential opportunities for meaningful interventions that may help close the diversity gap in medicine.

## Methods

### Study overview

We conducted a cross-sectional study of students at Stanford School of Medicine to examine belongingness amongst first-generation students. We collected survey data in August 2019 that we analyzed from 2020 through 2022.

### Population

Stanford School of Medicine in Palo Alto, California is a research-intensive school with a total enrollment of approximately 488 students. Over half of these students finish their studies in 5 or more years to pursue a second degree or conduct research. Stanford is an ideal setting for this study because the school attracts a diverse student body, has selection processes aimed at achieving class diversity across many domains, and boasts a comprehensive first-generation mentoring program. Eligible study participants included all students who had completed at least 1 year of medical school.

### Ethical considerations

We explained the risks of study participation in a solicitation letter and completion of the survey implied informed consent. We entered participants in a raffle for a $100 gift card as an incentive to complete the survey. The Stanford School of Medicine Institutional Review Board approved this study (protocol #51320).

### Survey instrument

We adapted Ingram’s survey tool on belongingness in college students, a modification of Hurtado and Carter’s original *General Sense of Belonging* tool ^18, 19^. We chose this instrument because of the potential to lend greater precision and clarity to the concept of belongingness by clearly differentiating and examining components of belonging and the relationships among them. The Ingram tool includes 23 items that encompass student belonging in four composites: *general belonging* (3 items), *social belonging* (“feeling socially comfortable and connected with peers as a member of the college community”; 8 items), *academic belonging* (“feeling respected and supported by faculty and in the classroom to do well academically”; 7 items), and *perceived institutional support* (“feeling that institutional support and student services are accessible on campus”; 5 items) ^18^. Ingram summarized the survey items, factor loadings, and Chronbach alphas for the instrument. Correlations among three composites of belonging ( *social belonging, academic belonging, and institutional support*) all have a *p*-value < 0.01 ^19^.

For the purposes of this study, we removed several items from Ingram’s tool to minimize survey fatigue. These included items in *social belonging* and *academic belonging* that each had the lowest factor loadings within their composites, and items in *social belonging* due to redundancy and a low correlation with belongingness. We combined *general belonging* and *social belonging*, as was done by Ingram ^19^. We deleted the word “women’s” from one item under *institutional support* to make the question relevant to students of all genders. The final instrument included 16 items grouped into 3 composites. As done by Ingram, we arranged survey responses to each item on a 5-point Likert scale: strongly disagree, somewhat disagree, neither agree nor disagree, somewhat agree, and strongly agree.

### Data collection and outcomes

We emailed all eligible participants an electronic survey designed using Qualtrics (Qualtrics Inc., Seattle, Washington) during a 4-week enrollment period. We sent reminders to non-respondents. We did not maintain individually identifying information and we did not solicit demographic information beyond the statuses described below to protect participant anonymity. A copy of the survey used can be found under *Extended data* ^20^ *.*

We examined all survey data using three participant outcomes: (1) *first-generation American status*, (2) *first-generation college status*, and (3) *first-generation graduate school status.*

### First-generation American status

“First generation” is sometimes defined by citizenship. For this study, we examined belongingness among participants who were in the first generation of their families to be born in the U.S. We asked students, “Are you a first-generation-born American or immigrant to the U.S.?” We categorized participants as either “Yes, first generation born in the U.S.”, “Yes, immigrant to the U.S.”, or “No”. We did not analyze data for immigrants to the U.S for this outcome because they did not meet our definition of first-generation American-born. This left two variables: “first-generation American” and “non-first-generation American.”

### First-generation College status

“First-generation” is sometimes based on whether a student’s parents attended college, so we examined belongingness among participants who were in the first generation of their families to attend college. We asked students to report the highest level of education attempted by any parent. We categorized participants as either “no college” if their parents did not pursue a college degree or “college” if at least one parent completed a college course of any kind anywhere in the world. We considered participants in the “no college” cohort to be “first-generation college students.”

### First-generation Graduate school status

Similarly, we examined belongingness based on a parent’s pursuit of a graduate degree. This is especially relevant in the context of medical school and belongingness as many students are the children of physicians, and those students are thought to have greater advantages in medical school because of their familiarity with the profession. Thus, we categorized participants as either “no graduate school” if their parents did not pursue graduate studies or “graduate school” if at least one parent completed a graduate course of any kind anywhere in the world. We considered participants in the “no graduate school” cohort to be “first-generation graduate school students.”

### Analysis

 For each of the three composite groups, we added corresponding item scores (Social [Q1-7], Academic [Q8-11], and Institutional [Q12-16]) into a single score for each individual participant. We then assessed the sums for differences between groups using a Mann-Whitney U-test. We did not use an item more than once in a composite group.

## Results

Of the 405 eligible participants, 82 (20.2%) completed the survey. Of these, 39% (32/82) identified as “first-generation American students,” 23.1% (19/82) identified as “first-generation college students,” and 41.4% (34/82) identified as “first-generation graduate school students.”

There were no significant differences observed between first-generation American students and non-first-generation American students for any of the individual survey items or the composite groups of items ( Table 1). However, first-generation college students scored significantly lower on 15 of the 16 items ( *p* < 0.05) and on all three composites of belonging ( *p* < 0.01) than participants whose parents had at least some college education ( Table 2). First-generation graduate school students also had significantly lower scores on 12 of the 16 items ( *p* < 0.05) and on all three composites of belonging ( *p* < 0.02) than students whose parents had at least some graduate school education ( Table 3). The full raw data can be found under *Underlying data* ^20^.

**Table 1.** Responses to each survey question and composite values by whether the respondent was the a first-generation American.

Survey items (Q1-16)	First-generation American status		*p* value ^b^
	First-gen *n* = 32	Non-first-gen *n* = 36	
Q1. I see myself as a part of the medical school community.	3.1 (1.0) ^a^	2.8 (1.2)	0.252
Q2. Students at this medical school are friendly with me.	3.3 (1.0)	3.4 (0.7)	0.945
Q3. I feel I belong at this medical school.	3.2 (1.0)	2.9 (1.2)	0.387
Q4. It has been easy for me to make friends in medical school.	2.9 (1.3)	2.4 (1.2)	0.056
Q5. I can really be myself at this medical school.	3.0 (1.2)	2.4 (1.4)	0.057
Q6. Other students in this school seem interested in my opinions, ideas, and questions related to coursework.	3.0 (1.1)	2.9 (1.0)	0.624
Q7. Other students here like me the way I am.	3.2 (1.1)	3.1 (0.7)	0.307
Q8. I feel comfortable asking a question in class.	2.8 (1.3)	2.9 (1.1)	0.99
Q9. I feel comfortable contributing to class discussions.	3.1 (1.1)	3.0 (1.1)	0.652
Q10. The professors here respect me.	3.4 (0.7)	3.3 (0.9)	0.844
Q11. I would feel comfortable asking a professor for help if I did not understand course-related material.	3.3 (1.0)	3.4 (0.9)	0.826
Q12. I can find resource center support services for individuals like me.	3.1 (1.0)	2.9 (1.1)	0.471
Q13. I can find counseling support services.	3.2 (1.1)	2.9 (1.2)	0.284
Q14. I can find career planning support services.	2.8 (1.2)	2.6 (1.3)	0.453
Q15. I can find tutoring support services.	2.9 (1.1)	2.8 (1.0)	0.704
Q16. I can find health and wellness support services.	3.2 (0.9)	2.8 (1.2)	0.181
**Composite measures**			
Social Belonging (Q1-7)	21.7 (7.0)	19.9 (3.0)	0.143
Academic Belonging (Q8-11)	12.7 (3.3)	12.6 (2.9)	0.708
Institutional Support (Q12-16)	15.2 (4.3)	14.0 (4.9)	0.255

^a^ Values are given as mean (standard deviation). Responses were on a scale of 1 (Strongly disagree) to 5 (Strongly agree) ^b^ Mann-Whitney U test. Red boxes indicate p<0.01, pink boxes indicate p<0.05.

**Table 2.** Responses to each survey question and composite values by whether the respondent was the first generation to attend college.

Survey items (Q1-16)	First-generation to college status		*p* value ^b^
	First-gen *n* = 19	Non-first-gen *n* = 63	
Q1. I see myself as a part of the medical school community.	2.4 (1.2) ^a^	3.0 (1.0)	0.02
Q2. Students at this medical school are friendly with me.	2.6 (.09)	3.5 (0.8)	0.0001
Q3. I feel I belong at this medical school.	2.1 (1.1)	3.2 (1.1)	0.0003
Q4. It has been easy for me to make friends in medical school.	1.6 (1.2)	2.8 (1.2)	0.0007
Q5. I can really be myself at this medical school.	1.7 (1.2)	2.8 (1.3)	0.0009
Q6. Other students in this school seem interested in my opinions, ideas, and questions related to coursework.	2.3 (1.0)	3.1 (1.1)	0.003
Q7. Other students here like me the way I am.	2.4 (0.8)	3.2 (0.9)	0.0001
Q8. I feel comfortable asking a question in class.	2.2 (1.4)	3.1 (1.1)	0.02
Q9. I feel comfortable contributing to class discussions.	2.7 (1.2)	3.2 (1.0)	0.07
Q10. The professors here respect me.	2.7 (1.0)	3.3 (0.8)	0.02
Q11. I would feel comfortable asking a professor for help if I did not understand course-related material.	2.6 (1.5)	3.4 (0.7)	0.04
Q12. I can find resource center support services for individuals like me.	2.3 (1.2)	3.0 (1.0)	0.02
Q13. I can find counseling support services.	2.3 (1.3)	3.2 (1.0)	0.002
Q14. I can find career planning support services.	2.1 (1.1)	2.8 (1.2)	0.01
Q15. I can find tutoring support services.	2.2 (1.3)	2.9 (1.0)	0.049
Q16. I can find health and wellness support services.	2.3 (1.4)	3.1 (1.0)	0.02
**Composite measures**			
Social Belonging (Q1-7)	15.1 (5.0)	21.6 (6.4)	0.0001
Academic Belonging (Q8-11)	10.3 (3.6)	13.0 (2.9)	0.003
Institutional Support (Q12-16)	11.2 (5.1)	15.0 (4.2)	0.003

^a^ Values are given as mean (standard deviation). Responses were on a scale of 1 (Strongly disagree) to 5 (Strongly agree) ^b^ Mann-Whitney U test. Red boxes indicate p<0.01, pink boxes indicate p<0.05.

**Table 3.** Responses to each survey question and composite values by whether the respondent was the first generation to attend graduate school.

Survey items (Q1-16)	First-generation to graduate school status		*p* value ^b^
	First-gen *n* = 34	Non-first-gen *n* = 48	
Q1. I see myself as a part of the medical school community.	2.5 (1.1) ^a^	3.2 (1.0)	0.0009
Q2. Students at this medical school are friendly with me.	2.9 (0.9)	3.5 (0.8)	0.0003
Q3. I feel I belong at this medical school.	2.4 (1.1)	3.3 (1.1)	0.0002
Q4. It has been easy for me to make friends in medical school.	2.0 (1.3)	2.9 (1.2)	0.0013
Q5. I can really be myself at this medical school.	2.0 (1.3)	3.0 (1.2)	0.0007
Q6. Other students in this school seem interested in my opinions, ideas, and questions related to coursework.	2.6 (1.1)	3.1 (1.0)	0.0126
Q7. Other students here like me the way I am.	2.6 (0.9)	3.4 (0.8)	0.0001
Q8. I feel comfortable asking a question in class.	2.5 (1.5)	3.1 (0.9)	0.0589
Q9. I feel comfortable contributing to class discussions.	2.7 (1.3)	3.3 (0.8)	0.0488
Q10. The professors here respect me.	3.0 (1.0)	3.4 (0.8)	0.0673
Q11. I would feel comfortable asking a professor for help if I did not understand course-related material.	2.9 (1.3)	3.5 (0.6)	0.0409
Q12. I can find resource center support services for individuals like me.	2.4 (1.3)	3.2 (0.9)	0.0061
Q13. I can find counseling support services.	2.7 (1.2)	3.2 (1.0)	0.0729
Q14. I can find career planning support services.	2.3 (1.2)	2.9 (1.2)	0.0299
Q15. I can find tutoring support services.	2.4 (1.1)	2.9 (1.0)	0.0222
Q16. I can find health and wellness support services.	2.7 (1.2)	3.1 (1.0)	0.2764
**Composite measures**			
Social Belonging (Q1-7)	16.9 (6.1)	22.4 (6.0)	0.0001
Academic Belonging (Q8-11)	11.1 (3.8)	13.3 (2.5)	0.0074
Institutional Support (Q12-16)	12.6 (4.8)	15.2 (4.3)	0.0129

^a^ Values are given as mean (standard deviation). Responses were on a scale of 1 (Strongly disagree) to 5 (Strongly agree) ^b^ Mann-Whitney U test. Red boxes indicate p<0.01, pink boxes indicate p<0.05.

## Discussion

Our findings indicate that parental education is strongly associated with belongingness among first-generation medical students. These results imply that parents who have had similar educational experiences might be able to better prepare and support their children for the unique stressors experienced in medical school. Interestingly, students who are first to attend college or graduate school had significantly lower belongingness scores on the social belonging and institutional support composites. This suggests that the capacity to experience meaningful relationships with peers and access adequate support through institutional resources play significant roles in determining belongingness in medical school. Surprisingly, we found no difference in belongingness based on a parent’s place of birth for students who were born in the U.S. This could indicate that students who grew up in the U.S. are comfortable within U.S. academic culture regardless of whether their parents were immigrants. 

Bronfenbrenner’s Environmental Satisfaction model offers an important lens with which to interpret these findings ^15^. This model suggests that belonging is determined by the degree of satisfaction one has with their interactions with others and their environment. First-generation students may be dissatisfied with interactions between themselves and their peers as illustrated by survey items that reflect perceived likeability (“Other students here like me the way I am.”) and interests in these students by others (“Other students in this school seem interested in my opinions, ideas, and questions related to coursework.”). Correspondingly, the Identity-Proximity model suggests that positive relationships with “other” groups determine belonging ^21^. First-generation students may feel inhibited by or be dissatisfied with classroom culture, specifically with respect to their comfort speaking up (“I feel comfortable asking a question in class.”) or asking for help (“I would feel comfortable asking a professor for help if I did not understand course-related material.”). This is unsurprising as agency in the classroom has been shown to increase belongingness ^21^.

Institutional support has also been associated with student belongingness in non-U.S. medical schools ^22^. Among our participants, first-generation students may experience barriers to access or be dissatisfied with available institutional resources, particularly career counseling (“I can find career planning support services.”) and tutoring resources (“I can find tutoring support services.”). Conversely, items regarding inclusive personal care (“I can find health and wellness support services.”) had little or no difference between groups. First-generation students knew how to access health services but found other services less accessible such as counseling, career services, or tutoring.

Importantly, we did not detect statistical difference for the item, “The professors here respect me.” This suggests that first-generation students feel respected in school even when they might not feel as though they belong. However, respect from faculty members alone is clearly insufficient when first-generation students experience poor relationships with peers, lack of agency in the classroom, and inadequate institutional support.

It is critical that first-generation students are adequately supported to meet the national demand for a more diverse physician workforce ^23^. Lack of belonging not only threatens the success of first-generation medical students but also their recruitment, wellness, and ongoing professional development ^24^. The implications are broad and involve numerous stakeholders in medical education that include faculty, administration, alumni, donors, and the students themselves. To further diversify medicine, targeted efforts to address social integration and identify the necessary support to facilitate belongingness amongst first-generation students is imperative.

### Potential interventions

Based on our findings and the theoretical frameworks we described, two types of interventions might improve belongingness: the first focuses on changing the individual’s perception of how they belong; and the second involves tailoring the social environment of the learner to stimulate belonging ^25^. For example, in a program called “Build & Belong,” senior medical students at one school created video messages about belongingness for junior medical students, who then wrote letters to future medical students after reflecting on the videos. This proved to decrease social isolation scores, which were associated with low belongingness, especially among Black medical students ^26^. Another intervention involved helping students reframe ambiguous experiences, such as constructive criticism, in a manner that encouraged a growth mindset instead of a fixed mindset ^27^. Furthermore, it can be helpful to have students focus on their in-group identities when feeling low levels of belonging instead of emphasizing their out-group identities ^28^. For medical students, Brown *et al.* created a social media handle, @FirstGenDocs, to facilitate an online community and enhance in-group identities for first-generation student physicians ^29^.

Positive educational experiences can improve medical student belongingness. Longitudinal exposure to teaching physicians during clinical rotations as a senior medical student improved the students’ “in-group fit” and sense of belonging during the transition from student to practitioner ^30, 31^. Longitudinal integrated clerkships in rural medicine increased students' senses of belonging in rural communities and their likelihood to practice there after training ^32^. More specifically, educational experiences in primary care increased students' senses of belonging and their choice to enter primary care specialties ^33^. Thus, optimizing the learning environment for medical students is especially important because their success and wellbeing is strongly associated with their professional identities and performance ^17^.

Coaching programs and guided reflective practices have been used successfully to improve student enculturation and social belonging in medical school ^34– 36^. Other interventions that change the social environment include students adopting a set of mutually agreed upon classroom goals and norms; ranking their priorities within the classroom; comparing their priorities to the teacher’s; supporting students while still allowing an appropriate sense of autonomy; and emphasizing dialogue within the learning environment ^25^.

Our study suggests that important interventions might include normalization of differences rather than “otherness” as well as intentionally cultivating an inclusive classroom environment ^37^. Identifying cause and effect could further inform the design of these interventions to enhance belonging. Research is needed to evaluate interventions for medical students, and interventions may need to be tailored differently for preclinical classrooms and clinical learning environments.

### Stanford School of Medicine

It is important to examine these findings in the historical and local contexts of the institution from which they were derived. Stanford School of Medicine provides robust student resources and programming that benefit first-generation students. The Stanford Leadership in Health Disparities Program helps students acculturate to new learning environments in medical school. More specifically, the First-Generation Mentorship Program at Stanford, established in 2015, is an optional year-long experience that matches first-generation students with first-generation mentors ^38^. The program offers community-building and educational activities including workshops that have addressed topics such as social belongingness, microaggressions and marginalization, and imposter syndrome. The program has 173 mentors (approximately 60% are Stanford alumni), of which 44 actively participate in monthly one-on-one mentoring, serve as program speakers, host events in their homes, or mentor optional scholarly projects. 79 medical school or graduate school students have completed the program. At the time of this study, the First-Generation Mentorship Program was in its fourth annual cycle and had addressed social belongingness in at least one panel discussion. Although it is not known whether any of the respondents participated in the First-Generation Mentorship Program, it could have affected some responses to the survey. Most likely this would have had the effect of making the differences between groups smaller.

Despite these resources, there were disparities in belongingness for first-generation students at Stanford School of Medicine, which gives us pause and opportunity for introspection. It is possible that not all first-generation students are being identified as they are referred to services based on information from their medical school applications. This indicates the need for more robust outreach to ensure all first-generation students have access to services. Information about the First-Generation Mentorship Program is provided during the school’s ‘second look’ weekend for admitted students and at medical school orientation. However, students are likely overwhelmed with announcements about similar school resources at those times. Information overload and consequential lack of familiarity with many specific institutional resources is supported by the findings of this study. To improve outreach efforts, a partnership with the Career Center has been planned to create more deliberate programming for first-generation students such as mock interviews, career counseling, and a Med Wiki to warehouse program resources. New preclinical clerkship offerings from the Stanford Office of Diversity in Medical Education will pay specific attention to the needs of first-generation students. Finally, a first-generation and/or low-income student interest group for medical students was established in 2020 that provides student-only programming related to advocacy and education. This program may have affected student belongingness after the time of data collection for this study.

### Limitations

There are several important limitations to these findings. Notably, the Ingram survey instrument has only been published in dissertation form, but it has been cited and used in a number of other studies and, thus, appears to be an accepted method in this field. Additionally, we modified the Ingram survey instrument by removing questions to reduce survey fatigue. We limited the effect of these changes by removing questions with low factor loading or low correlation with belongingness. However, we did not perform an independent analysis of the validity of the modified instrument. There is also risk of response biases, as with all survey studies, such as extreme response bias, acquiescence bias, and social desirability bias. These may have affected the results given the personal and potential emotional nature of the survey content.

Lastly, we conducted this study at only one institution, and it is limited by a response rate of 20%, which affects the generalizability of these findings to other medical schools. We also chose not to control for other demographic factors (e.g., socioeconomic status, race, ethnicity, gender, sexuality, disability status) in an effort to preserve anonymity due to the small number of expected participants. There is a need for larger investigations across many schools to properly identify disparities in belongingness between medical student populations. Qualitative studies may better uncover the sources of disparities and describe in more depth how they impact students during medical school.

## Conclusion

Medical students who are first in their families to attend college or medical school report lower belongingness than their peers. Peer relationships and institutional support play an important role in belongingness. To meet the need for a more diverse physician workforce, interventions must be designed to better address belongingness for these students. Ongoing efforts to assess belongingness, the effectiveness of existing interventions, and quality of innovations and improvements are warranted.

## Data Availability

Zenodo: Belongingness among first-generation medical students. https://doi.org/10.5281/zenodo.10052439 ^20^ firstGen_survey_rawData_redactedFinal.xlsx firstGen_survey_key.txt Data are available under the terms of the Creative Commons Attribution 4.0 International license (CC-BY 4.0).
