# Supplementary material for: Belongingness among first-generation students at Stanford School of Medicine
Source: MedEdPublish (2016). 2023 Nov 23;13:288. [Version 1] doi: 10.12688/mep.19912.1 (PMC11061589; doi:10.12688/mep.19912.1)
Supplement: Peer review history

# Reviewer report: Reviewer response for version 1

**DOI:** 10.21956/mep.21328.r36124

**Published:** 2024-04-30

**Authors:** Virginia Sheffield, Catherine McDermott

**Affiliations:** 1 University of Michigan, Ann Arbor, Michigan, USA

**Summary of the Article**

 This study examines the relationship between first-generation student status and feelings of belongingness at Stanford School of Medicine. Using a modified survey instrument, the authors find that first-generation students report lower levels of belongingness, indicating the need for targeted interventions to support this group.

**Article Relevance**

 With shifting demographics in medical schools, and a growing number of studies emphasizing the unique challenges faced by minoritized students, this is important work to fill gaps in literature surrounding belongness particularly for those who are historically underrepresented such as first-generation students. Understanding this topic can be leveraged to work to create a more equitable and inclusive learning environment.

**Methodology**

 The methodology of this article is sound. The modifications to the validated survey are logical for the context in which it was used. The response rate of 20% is low, however still provides meaningful data. Lack of ability to correlate findings with any other demographic data is a significant limitation in this study.

**Specific Points of Feedback**

- Because demographic data was not collected, there is major limitation in being able to interpret the findings of this survey. The discussion would benefit from more specific details about what prior literature has shown in terms of demographics of first-generation students (making certain to differentiate first generation in medicine as a whole vs the authors’ definition of having a parent who ever took any single graduate school class). The discussion should make sure to note how intersecting identities of first-generation students might impact the results.
- In the discussion, you note the following: “Our findings indicate that parental education is strongly associated with belongingness among first-generation medical students. These results imply that parents who have had similar educational experiences might be able to better prepare and support their children for the unique stressors experienced in medical school.” Based on your survey items, I am not sure that you can draw these conclusions. Your data supports that higher parental education is associated with higher levels of belonging but does not provide insight into whether that is because their parent’s education allows them to better prepare or support their children for the unique stressor of medical school. I would also argue that it is unclear whether these parents’ educational experiences were similar (a parent who took one course at a community college would be very different to the experience of medical school).

- You also note, “This suggests that the capacity to experience meaningful relationships with peers and access adequate support through institutional resources play significant roles in determining belongingness in medical school.” Your survey questions explore one’s abilities though not capacity. This conflates skill with potential.
- The first sentence of your conclusion reads “medical students who are first in their families to attend college or medical school report lower belongingness than their peers.” However, your data did not stratify those who were first in their family to go to medical school, only graduate school.

- While the study focuses on first generation students, much of the introduction focuses on the importance of achieving greater diversity in the physician workforce though does not delineate how first-generation students contribute to this diversity. I would consider expanding on this. Particularly expanding on any literature that gives data on the demographics of first-generation students.

- In the introduction, you note that studies show that first-generation students lack a sophisticated knowledge of the medical school “admissions game.” More information about what you mean by “admissions game” would be helpful. Additionally, the articles you cite to support this are all out of the UK though your study is focused on American medical students. Perhaps exploring the literature on hidden curriculum and URiM students would yield more support to your argument.
- Please define first generation medical student (first student to attend medical school, to attend higher education, etc.) and ensure that your use of the term matches that in the literature that you cite.
- In the introduction, you mention that those underrepresented in medicine include first-generation medical students which is discordant with the AAMC definition (racial and ethnic populations that are underrepresented in the medical profession relative to their numbers in the general population). Subsequently you discuss first-generation American, college, and graduate school though do not discuss first generation medical student.
- I appreciate the discussion on the importance of positive opportunities (especially positive longitudinal opportunities) and how this increases a sense of in-group fit. However, you transitioned the discussion to how working in a rural community increased the sense of belonging in that community, which, while important, does not necessarily correlate with a student’s sense of belonging in their medical school. Similarly, I was confused about your discussion about the desire to enter primary care and how this related to one’s sense of belonging in medical school.

Have any limitations of the research been acknowledged?

Yes

Is the work clearly and accurately presented and does it cite the current literature?

Yes

If applicable, is the statistical analysis and its interpretation appropriate?

I cannot comment. A qualified statistician is required.

Are all the source data underlying the results available to ensure full reproducibility?

Yes

Is the study design appropriate and is the work technically sound?

Yes

Are the conclusions drawn adequately supported by the results?

Partly

Are sufficient details of methods and analysis provided to allow replication by others?

Yes

Reviewer Expertise:

bias in assessment, lactation support for trainees, responding to patient initiated harassment

We confirm that we have read this submission and believe that we have an appropriate level of expertise to confirm that it is of an acceptable scientific standard.

# Reviewer report: Reviewer response for version 1

**DOI:** 10.21956/mep.21328.r36121

**Published:** 2024-04-08

**Authors:** David Kirtchuk, Francesca Hall

**Affiliations:** 1 Brighton And Sussex Medical School, Brighton, UK; 2 BSMS, Brighton and Sussex Medical School, Brighton, Sussex, UK

I applaud the work here which is necessary as medical education literature needs to explore how belongingness is experienced in diverse student groups.  

 I believe this article should be approved with reservations.

1. There are a few areas of the paper that could benefit from greater exploration, whilst also using language that reflects the limitations of this type of research. This is particularly the case when it comes to understanding cause and effects of the findings.  
2. Grouping students based on a single attribute is complicated and does not mean the students are homogenous in nature. A lack of a demographic breakdown of both groups makes any assumptions from the findings challenging to interpret if one is attempting to identify the drivers of a sense of lack of belonging. This raises the issue of confounders as no other demographics are controlled for and thus extrapolating cause and effect of the differences identified in the paper are limited.   
3. There is a large pool of existing literature regarding student belongingness and we feel more could be done in the discussion portion to explore where these findings sit within the literature. For example, in the discussion segment a comment is made regarding a strong association with parental education and belonging.  Whilst this may be true I think it is hard to be certain what factors drive this.  A note should be made in the limitations to make this explicit.  We are not convinced your findings **imply** that parental education status is directly linked to belonging as these parents are less well placed to support their children. Belongingness is far more complex and nuanced an issue.  Is parental education a proxy for class/financial/cultural capital?
4. There could be some attention given to intersectionality and how it influences the issues you discuss. By removing the term ‘woman’ from your survey instrument, there is a lost opportunity to see if students in that particular group have a different experience. Consider Bourdieu’s theories regarding this as a potential source to explore? The work of Costello may offer some understanding within your cultural context.
5. The paper opens by describing Stanford as attracting a ‘diverse student body’. However, it is internationally recognised as an elite institution. Later in in paper, a considerable part of the article is devoted to explaining to the support that students could expect to receive as a first generation student. There is a slight sense of discord between your suggestions that Stanford has a diverse population, but that support is required.
6. Therefore, of the un examined social characteristics acknowledged in the paper, I would suggest social class to be a key missing possibly explanatory factor influencing ‘belongingness’. Reay and others consider this in from a UK perspective.[1]
7. We believe the major conclusion that can be drawn from the findings in this paper is that a difference exists but the study was not designed to be able to explore this in greater detail. Therefore, discussion statements need to consider the degree of extrapolation drawn from the results.

There are a few minor points that could be clarified.

1. Are students who responded to the survey over-representative of first generation students compared to the overall cohort of students at the medical school? Is there data held by the admissions team at the school to compare with? This would help understand whether the results are more likely to be biased?
2. Were there differences across year groups? Pre-clinical vs clinical attachments? Or was there insufficient data to extrapolate this? There are a number of groups that students are expected to belong to during the course of becoming a doctor; higher education student, medical student and doctor amongst others. These identities change over time and ‘belongingness’ to one group may not signify the same sense to all groups.
3. Results – The comparator group is a bit unclear in the results section. Table 1 numbers add up to 68 (what happened to the other 14 respondents? Why were they not included? Was this because they met the criteria for first generation students but not based on parent immigration?)
4. How much cross over is there between the 3 groups of first generational students? How does that likely impact the findings?
5. We think additional comments regarding the need to understand what is meant by belonging and what this does to students from first in family populations. The suggestions for potential interventions seem to focus on how to make students belong but don’t consider the need for medical institutes to reflect on the learning environment and culture that exists and how their policies contribute to it. I.e. the balance between students from diverse backgrounds having to adapt to belong and the degree to which the institutional culture needs to develop and adapt.
6. We wonder whether the section which describes what Stanford does to support students could instead be uses to orientate the research within the existing literature as that would provide much needed context for the results.

Have any limitations of the research been acknowledged?

Partly

Is the work clearly and accurately presented and does it cite the current literature?

Partly

If applicable, is the statistical analysis and its interpretation appropriate?

Partly

Are all the source data underlying the results available to ensure full reproducibility?

Yes

Is the study design appropriate and is the work technically sound?

Yes

Are the conclusions drawn adequately supported by the results?

Partly

Are sufficient details of methods and analysis provided to allow replication by others?

Yes

Reviewer Expertise:

Assessment, widening participation, remediation & support, student belonging, identity formation

We confirm that we have read this submission and believe that we have an appropriate level of expertise to confirm that it is of an acceptable scientific standard, however we have significant reservations, as outlined above.

## References

[1] : ‘Fitting in’ or ‘standing out’: Working‐class students in UK higher education. *British Educational Research Journal* .2010;36(1) : 10.1080/01411920902878925 107-124 10.1080/01411920902878925

# Reviewer report: Reviewer response for version 1

**DOI:** 10.21956/mep.21328.r36126

**Published:** 2024-03-28

**Authors:** Charmaine Troy

**Affiliations:** 1 Georgia Tech University, Atlanta, GA, USA

Thank you for the opportunity to review this article. I applaud the authors for writing this article about the importance of belongingness among first-generation students in medical school. It is a topic that is not widely covered in the literature and I am excited to see it. Sense of belonging is a key factor in the success of a first-generation student’s experience, both inside and outside of the classroom. I recommend approval of this article with revisions. I have a couple of concerns that can be addressed in a revision. Below are my concerns:

**Major Concerns:**

1. I would like to see more detail in this article about their mentoring program at Stanford. I don’t think that it gives enough context about the program and how it can contribute to the importance of the study. Some of the detail in this section could include:
  1. Program Description
  2. Brief background and data on the first-generation medical students at Stanford
  3. Mission, goals, and objectives of program
  4. Brief discussion about program implementation
  5. Stakeholders
  6. How assessment/metrics of belongingness was collected
  7. Outcomes
  8. Lessons learned

Minor Concerns:

1. There was a reference to the social media handle @FirstGenDocs. The #FirstGenDocs online community and social media movement was created by Lamesha Brown, Raven Cokeley, and Jason Wallace. This community wasn’t created for medical students specifically. It was created to serve and create community for first-generation Ph.D. students. This information should either be updated in the article or removed. Consider using the IG account “FoosinMedicine” instead. Very popular social media handle that follows two first-generation students in medical school.
2. Consider adding some data/metrics to your examples in the potential interventions section of the article. For example, the article mentions that educational experience in primary care increased students’ sense of belonging and their choice to enter primary specialties. By what percentage did it increase?
3. In the Stanford School of Medicine section, it discussed the first-generation student interest group of medical students and its possible impact on student belongingness but wasn’t sure of this and didn’t include concrete data or information. You may want to consider removing this or providing some metrics about the impact.

Have any limitations of the research been acknowledged?

Yes

Is the work clearly and accurately presented and does it cite the current literature?

Yes

If applicable, is the statistical analysis and its interpretation appropriate?

Yes

Are all the source data underlying the results available to ensure full reproducibility?

Yes

Is the study design appropriate and is the work technically sound?

Yes

Are the conclusions drawn adequately supported by the results?

Partly

Are sufficient details of methods and analysis provided to allow replication by others?

Yes

Reviewer Expertise:

First-Generation College Access and Success, First-Generation Student Mental Health and Wellness, Development and Implementation of First-Generation Programming, Education Access and Success for Rural Black First-Generation Students, Intersectionality and the First-Generation Student Identity

I confirm that I have read this submission and believe that I have an appropriate level of expertise to confirm that it is of an acceptable scientific standard, however I have significant reservations, as outlined above.

# Reviewer report: Reviewer response for version 1

**DOI:** 10.21956/mep.21328.r35364

**Published:** 2023-12-14

**Authors:** Michael Soh

**Affiliations:** 1 Uniformed Services University of the Health Sciences, Bethesda,, MD, USA

Thank you for the opportunity to review this manuscript. I commend the authors for engaging in this work as wellbeing, belongingness, and academic performance – among many other factors - are critical elements for success in medical school. While I recommend approval for this manuscript, I have some concerns that I believe can be addressed in a potential revision. I’ve noted them below, divided into major and minor concerns:

 Major Concerns

  

1. The premise of the front-end relies on recruiting medical students from diverse backgrounds so they can then go on to practice in underserved areas. Is this not the responsibility of all medical students? I find it problematic that underrepresented medical students, if you will, are the “fix” for serving and practicing in areas that are arguably the most challenging, most under-resourced, least retained, and least compensated?
2. Could there be differences between the classes at Stanford? In other words, could an MS4 report on belongingness differently than an MS2? I imagine the latter’s experiences are a bit more intense since everything is relatively new and they haven’t yet experienced clerkships. Moreover, an MS3 may likely experience belongingness much differently than an MS2 given the vast literature on the clerkship experience. I say all this to question whether it would be worth disaggregating the sample by cohort.
3. I think there are serious concerns about the tweaks made to the instrument. It is unclear how this impacts validity evidence of the instrument. By combining factors and removing single items, it is difficult to determine if and how these constructs still measure for the intended domains. This deserves a bit more real estate in the limitations and perhaps in the results (as a caveat to the findings). Additionally, is there a case to interpret individual items (versus the constructs)? If so, does removing items from the instrument impact this interpretation?
4. Are first generation Americans, first generation college students, and first generation graduate students one and the same? I suspect they are different and nuanced but it seems like the manuscript combines all of these together as one group. The front end seems to refer to first generation college students and I wonder if the authors can expound on if, and how, first generation college students might differ from Americans and graduate students?
5. I also have concerns about the response rate and the sample size of some of the groups reported (comparing 19 vs 63 in Table 2). Perhaps the authors can include power indices and/or effect sizes?
6. Of the respondents, how many reported being a first generation American, a first generation college student, AND a first generation graduate student? I’m wondering if there’s any sort of compounding effect here. In other words, does being first generation for each of these phases mitigate negative effects of belongingness? Or somehow exacerbate them?

Minor Concerns

  

1. The authors speak to the lack of statistical significance of the professor-respect item. However, the tables indicate that these items trend lower for the first-gen group. I wonder if it might be worth softening the language here and speaking to the trends being observed. In other words, with more power, could these differences have been significant?
2. Belongingness does not occur in a vacuum and I was hoping to read more about how this weaves into existing literature about professional identity formation and wellbeing. The authors nod very briefly to this when speaking to possible interventions but I believe this deserves a bit more focus when stepping back and interpreting these findings in the bigger picture.

Have any limitations of the research been acknowledged?

Yes

Is the work clearly and accurately presented and does it cite the current literature?

Yes

If applicable, is the statistical analysis and its interpretation appropriate?

Yes

Are all the source data underlying the results available to ensure full reproducibility?

Yes

Is the study design appropriate and is the work technically sound?

Yes

Are the conclusions drawn adequately supported by the results?

Partly

Are sufficient details of methods and analysis provided to allow replication by others?

Yes

Reviewer Expertise:

Health professions education; wellbeing; assessment and validity

I confirm that I have read this submission and believe that I have an appropriate level of expertise to confirm that it is of an acceptable scientific standard, however I have significant reservations, as outlined above.
